# CE‐MS for metabolomics: Developments and applications in the period 2016–2018

**DOI:** 10.1002/elps.201800323

**Published:** 2018-10-01

**Authors:** Rawi Ramautar, Govert W. Somsen, Gerhardus J. de Jong

**Affiliations:** ^1^ Biomedical Microscale Analytics Leiden Academic Center for Drug Research Leiden University Leiden The Netherlands; ^2^ Division of BioAnalytical Chemistry Amsterdam Institute for Molecules, Medicines and Systems Vrije Universiteit Amsterdam Amsterdam The Netherlands; ^3^ Biomolecular Analysis Utrecht Institute for Pharmaceutical Sciences Utrecht University Utrecht The Netherlands

**Keywords:** Biomedical and clinical, Mass spectrometry, Metabolomics, Microbial and plant, Technological developments

## Abstract

In the field of metabolomics, CE‐MS is now recognized as a strong analytical technique for the analysis of (highly) polar and charged metabolites in a wide range of biological samples. Over the past few years, significant attention has been paid to the design and improvement of CE‐MS approaches for (large‐scale) metabolic profiling studies and for establishing protocols in order to further expand the role of CE‐MS in metabolomics. In this paper, which is a follow‐up of a previous review paper covering the years 2014–2016 (*Electrophoresis* 2017, 38, 190–202), main advances in CE‐MS approaches for metabolomics studies are outlined covering the literature from July 2016 to June 2018. Aspects like developments in interfacing designs and data analysis tools for improving the performance of CE‐MS for metabolomics are discussed. Representative examples highlight the utility of CE‐MS in the fields of biomedical, clinical, microbial, and plant metabolomics. A complete overview of recent CE‐MS‐based metabolomics studies is given in a table, which provides information on sample type and pretreatment, capillary coatings and MS detection mode. Finally, some general conclusions and perspectives are given.

**Abbreviations**
ALS: amyotrophic lateral sclerosis
CF: cystic fibrosis
MSI: multisegment injection
QC: quality control

## 1 Introduction

The field of metabolomics has developed significantly over the past decade and basically progressed from a (fundamental) research topic studied by a relatively small number of highly specialized research groups into a major field now used by hundreds of laboratories, core facilities, and national centers 1. Currently, the two main techniques employed for (global) metabolic profiling studies are MS hyphenated to LC and NMR spectroscopy. Within the metabolomics field, CE‐MS especially emerged as a useful analytical technique for the profiling of (highly) polar and charged metabolites 2, 3, 4, 5, 6.

Until now, CE‐MS has only been used by a limited number of research groups for metabolomics studies. The coupling of CE to MS is still perceived as technically challenging by the scientific community and there is a lack of standard operating procedures, which are critical for performing (long‐term and interlaboratory) reproducibility studies. However, a number of recent studies clearly exemplify the usefulness of CE‐MS for metabolic profiling of large sample sets 7, 8, 9. For example, the group of Soga and coworkers, the group that introduced the first CE‐MS methods for metabolomics in 2003, has assessed the long‐term performance of CE‐MS for metabolic profiling of more than 8000 human plasma samples from the Tsuruoka Metabolomics Cohort Study over a 52‐month period 7. The study provided an absolute quantification for 94 polar metabolites in plasma with a similar or better reproducibility when compared to other analytical platforms, i.e. reversed‐phase LC‐MS and GC‐MS, employed for large‐scale metabolomics studies. The CE‐MS approach used for this large‐scale metabolomics study was provided by Human Metabolome Technologies (HMT), a company resulting from the first CE‐MS‐based metabolomics work of Soga and coworkers at Keio University 10. Although the CE‐MS approach of HMT for cationic metabolic profiling at low‐pH separation conditions employing a fused‐silica capillary can be used in a robust way and currently employed by various research groups, the development of a robust CE‐MS approach for anionic metabolic profiling has been and still is an active area of research 10, 11, 12, 13, 14, 15, 16, 17. For example, Yamamoto et al. has recently shown that commonly used ammonium acetate or ammonium formate BGEs with a pH above 9.0 can contribute to incidental capillary fractures via irreversible aminolysis of the outer polyimide coating 18. Prevention of polyimide aminolysis could be easily achieved by using weakly alkaline, ammonia containing buffers (pH < 9.0).

The previous example clearly underscores the need for well‐documented and detailed experimental procedures concerning the development of reliable and robust CE‐MS approaches for metabolomics. In this context, there appears to be a gradual effort among the CE‐MS community to highlight relevant methodological aspects for metabolomics studies in protocol papers and to share experimental procedures via peer‐reviewed video articles 19, 20, 21, 22, 23, 24, 25, 26. Apparently, such work is also needed to convince the scientific community about the unique and complementary capabilities of CE‐MS for metabolomics.

Though CE‐MS employing a coaxial sheath‐liquid interface for the hyphenation is most frequently used for metabolomics studies, recent developments in interfacing techniques enabled new applications due to the improved detection sensitivity that can be provided by such designs 27. In this paper, which is a follow‐up of our previous CE‐MS‐based metabolomics reviews 28, 29, 30, 31, 32, an overview of the latest advancements in CE‐MS approaches for metabolomics is provided as reported over the past two years. Attention will be paid to main technological developments, including new interfacing designs and use of improved CE‐MS conditions for further enhancing the metabolic coverage. Also new strategies for metabolite identification by CE‐MS will be outlined. The recent CE‐MS‐based metabolomics studies are summarized in a table and selected representative examples will be highlighted in order to show the utility of CE‐MS in the fields of biomedical, clinical, microbial, and plant metabolomics. Finally, some general conclusions and perspectives are provided.

## 2 Technological developments

**Figure 1:**
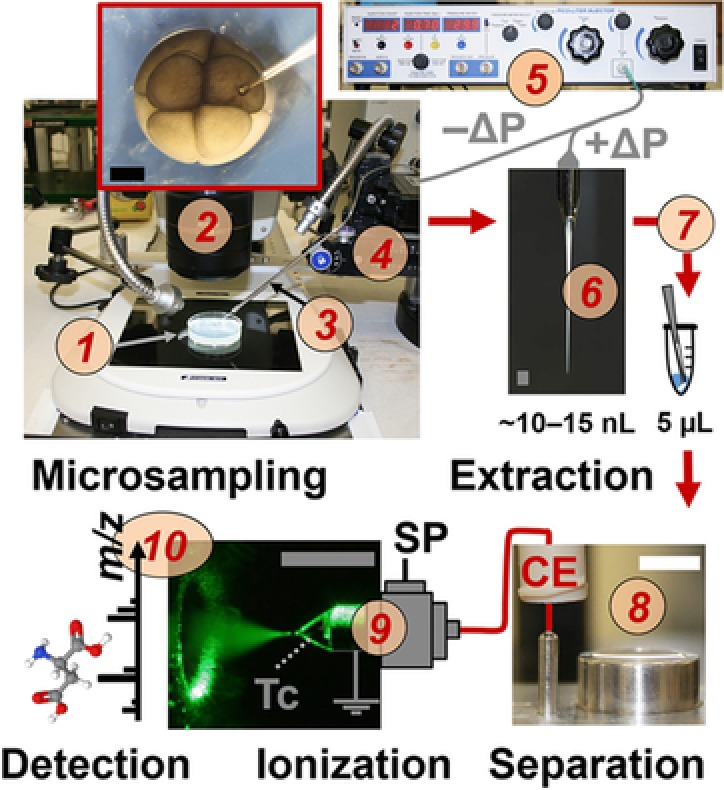
Microprobe single‐cell CE‐MS enabling in situ metabolic characterization of live *Xenopus laevis* embryos (1). A 10–15 nL portion of single cells identified under a stereomicroscope (2) were aspirated into a pulled capillary (3) using a multiaxis translation stage (4) and a micro‐injector (5) delivering vacuum (−ΔP). The collected cell content (6) was pressure‐injected (+ΔP) into a vial for metabolite extraction (7). The extract was measured by a microloading CE platform (8) connected to a CE‐ESI source (9) operated in the cone‐jet mode (see Taylor‐cone, Tc). Metabolite ions were identified and quantified using a high‐resolution MS instrument (10). Scale bars = 500 μm (dark/gray); 10 mm (white). SP, syringe pump. Reproduced from 33 with permission.

A major analytical challenge in metabolomics is still the profiling of polar and charged metabolites in limited amounts of sample material. In order to allow the analysis of metabolites in single cells, Onjiko et al. developed a microprobe single‐cell CE‐MS approach, in which microsampling, metabolite extraction and CE‐MS analysis were integrated as one analytical workflow (Fig. 1) 33. With this approach a small portion (10–15 nL) of the cell content from the *Xenopus laevis* embryo could be collected employing micropipettes based on borosilicate capillaries. For metabolite extraction, the 10–15 nL aspirate was expelled in a microvial containing 4 μL of extraction solvent, which was composed of 40% v/v acetonitrile and 40% v/v methanol at 4°C. After centrifugation, 10 nL of the supernatant was injected into a CE‐MS system, which used a custom‐built microflow sheath‐liquid interface. In this configuration the sheath‐liquid was provided at a flow‐rate of 1 μL/min. For more experimental details on the experimental workflow the reader is referred to a published video protocol 24. Overall, microprobe CE‐MS of less than 0.02% of the single‐cell content allowed the detection of circa 230 different metabolite features (in positive ion mode), including 70 known metabolites, in single dorsal and ventral cells in 8‐to‐32‐cell embryos. Relative quantification followed by multivariate and statistical analysis of the data revealed that microsampling improved detection sensitivity as compared to whole‐cell dissection by minimizing chemical interferences and ion suppression effects from the culture media.

**Figure 2:**
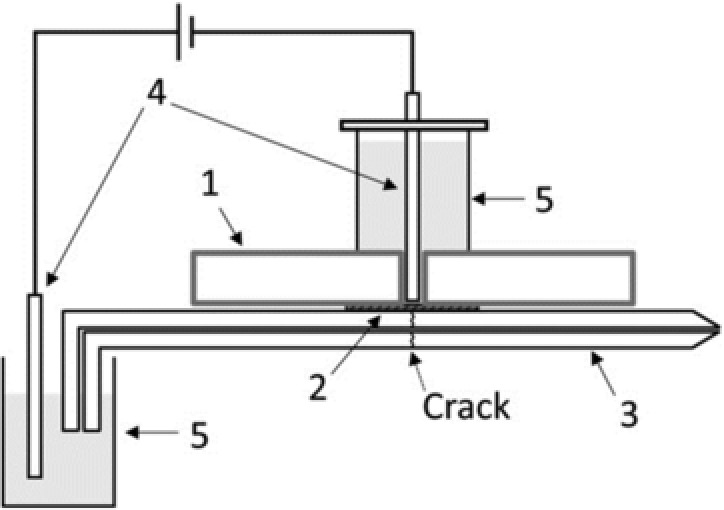
Schematic of the sheathless CE‐ESI‐MS interface: (1) plastic plate (2 mm thick); (2) electrodialysis membrane; (3) separation capillary; (4) platinum electrode; and (5) buffer reservoir. Reproduced from 34 with permission.

**Figure 3:**
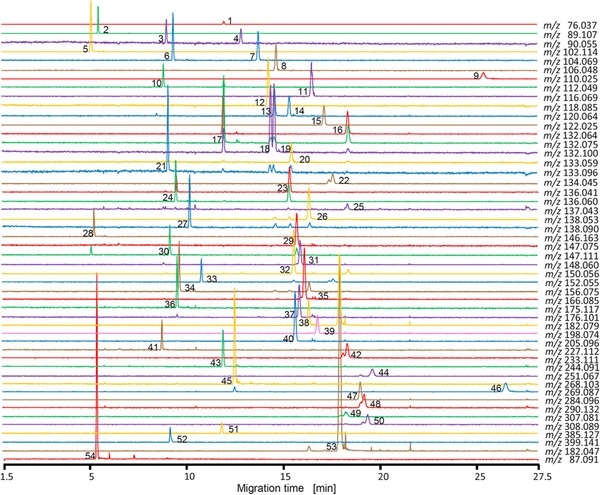
Extraction ion electropherograms obtained for the analysis of 52 cationic metabolite standards by sheathless CE‐MS. Peak identification: (1) glycine; (2) putrescine; (3) β‐alanine; (4) alanine; (5) spermine (divalent); (6) γ‐aminobutyric acid; (7) 2‐aminobutyric acid; (8) serine; (9) hypotaurine; (10) cytosine; (11) proline; (12) valine; (13) homoserine; (14) threonine; (15) cysteine; (16) hydroxyproline; (17) creatine; (18) isoleucine; (19) leucine; (20) asparagine; (21) ornithine; (22) aspartic acid; (23) homocysteine; (24) adenine; (25) hypoxanthine; (26) anthranilic acid; (27) tyramine; (28) spermidine; (29) glutamine; (30) lysine; (31) glutamic acid; (32) methionine; (33) guanine; (34) histidine; (35) phenylalanine; (36) arginine; (37) citrulline; (38) tyrosine; (39) 3,4‐dihydroxyphenylalanine; (40) tryptophan; (41) carnosine; (42) γ‐glutamyl‐2‐aminobutyric acid; (43) cytidine; (44) γ‐glutamyl‐cysteine; (45) adenosine; (46) inosine; (47) guanosine; (48) ophthalmic acid; (49) oxidized glutathione (divalent); (50) reduced glutathione; (51) S‐adenosylhomocysteine; (52) S‐adenosylmethionine; (53) methionine sulfone (internal standard); and (54) 3‐aminopyrrolidine (internal standard). Experimental conditions: standard concentrations, 20 μmol/L each; internal standard concentrations, 200 μmol/L each. A bare fused‐silica capillary (30 μm ID) used for electrophoretic separation employing 10% acetic acid as BGE at +30 kV. Samples were injected at 5 kPa for 15 s. Reproduced from 34 with permission.

The sheath‐liquid interface is generally used for CE‐MS‐based metabolomics studies; however, the CE effluent is significantly diluted in such a design thereby compromising the detection sensitivity. Therefore, Hirayama et al. developed a new sheathless interface for coupling CE to MS 34. This interface was designed by creating a small crack approximately 2 cm from the end of the capillary (Fig. 2), which part was covered with an electrodialysis membrane (cellulose acetate, molecular weight cut‐off of 100 Da) to minimize the migration of metabolites across the crack. An advantage of the proposed sheathless interface design is that it can be used with any commercially available capillary. However, it was found that S/N‐ratios for most cationic metabolite standards were comparable or higher with the use of a 30 μm id capillary (1.4 nL injected) as compared to a 50 μm ID capillary (13 nL injected), due to a higher background noise with the latter capillary. The use of 20 μm id capillaries resulted in unstable electrospray formation, therefore, 30 μm id capillaries were considered as optimal for this interfacing design. The performance of the sheathless CE‐MS method was assessed by analyzing a representative cationic metabolite mixture (Fig. 3). An injection volume of circa 1.4 nL provided LODs values for the test compounds in the range of 30–1000 nM, which were comparable with results obtained by sheathless CE‐MS using a porous tip interface. For test compounds, RSD (*n* = 3) values for migration times and peak areas (corrected with internal standard) were below 2.4% and 16%, respectively. Compared to sheath‐liquid CE‐MS employing the same capillary dimension and separation conditions, a 4.4‐fold improvement in LODs for the test compounds were obtained by this sheathless CE‐MS design. The method was used for the profiling of cationic metabolites in a limited amount of cancer cells.

**Figure 4:**
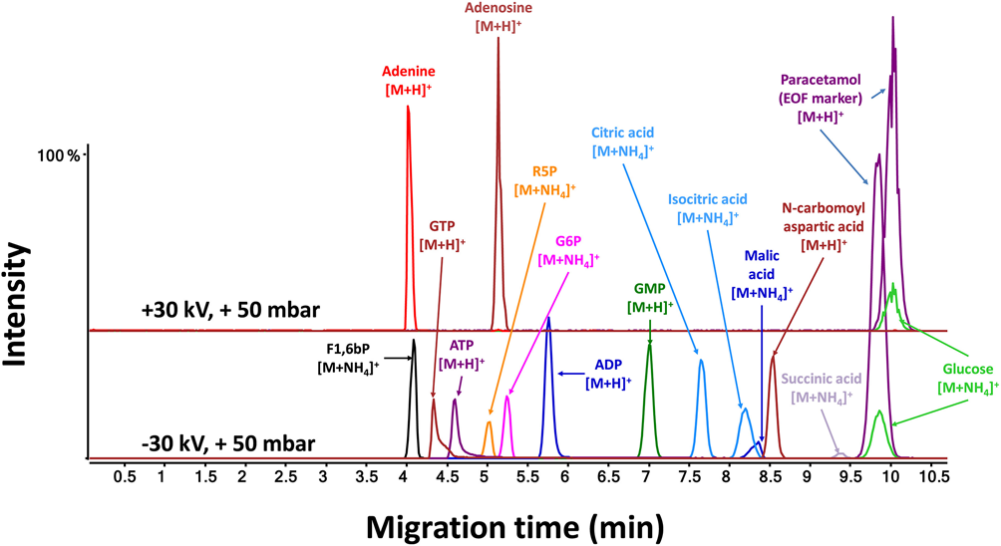
Extracted ion electropherograms obtained for a metabolite test mixture by CE‐MS in positive ion mode. Electrophoretic separation performed at low‐pH separation conditions using 10% acetic acid as BGE. The nebulizer gas was set to 0 psi, sheath gas was set to 11 L/min and ESI was performed at a voltage of 5500 V. The sheath‐liquid was composed of acetic acid (5 mM) and ammonium hydroxide (5 mM) in an isopropanol‐water (1:1, v/v) solution and delivered at a flow‐rate of 3 μL/min. Reproduced from 35 with permission.

In a conventional CE‐MS approach for metabolomics, the coaxial sheath‐liquid interface is used with a nebulizing gas. However, the use of a nebulizing gas may cause suction effects resulting in peak broadening and lower detection sensitivities. Therefore, Drouin et al. assessed the performance of CE‐MS employing the sheath‐liquid interface without nebulizing gas for a cationic metabolite mixture (including some basic drugs) using a design‐of‐experiments approach 35. Apart from setting the nebulizing gas to 0 psi, the sheath gas was set to 11 L/min at a temperature of 150°C. Moreover the capillary voltage was increased to 5500 V. Elimination of nebulizing gas resulted in a slightly increased analysis time and improved separation efficiency. For most test compounds, the S/N‐ratio significantly improved when using the optimized source conditions. The advanced CE‐MS approach has been used for the profiling of both anionic and cationic metabolites employing one single capillary and buffer combination. For the electrophoretic separation, 10% acetic acid was selected as BGE on the basis of the work of Gulersonmez et al. who has developed a sheathless CE‐MS approach for metabolic profiling of extracts from a glioblastoma cell line 17. In contrast to the latter work, anionic metabolites were detected by MS in positive ion mode as the presence of ammonium in the sheath‐liquid allowed the formation of ammonium adducts. Therefore, both basic and (many) acidic metabolites could be analysed by CE‐MS in the positive ion mode, as shown in Fig. 4. The overall approach significantly improved the metabolic coverage. When applied to the analysis of a commercially available metabolite library mixture comprising 596 compounds, more than 76% could be detected by the improved CE‐MS approach. A comparison with HILIC‐MS and reversed‐phase LC‐MS revealed that CE‐MS is particularly well‐suited for the profiling of amino acids, sulphated, and phosphorylated compounds, thereby clearly illustrating the added value of CE‐MS for metabolomics.

**Figure 5:**
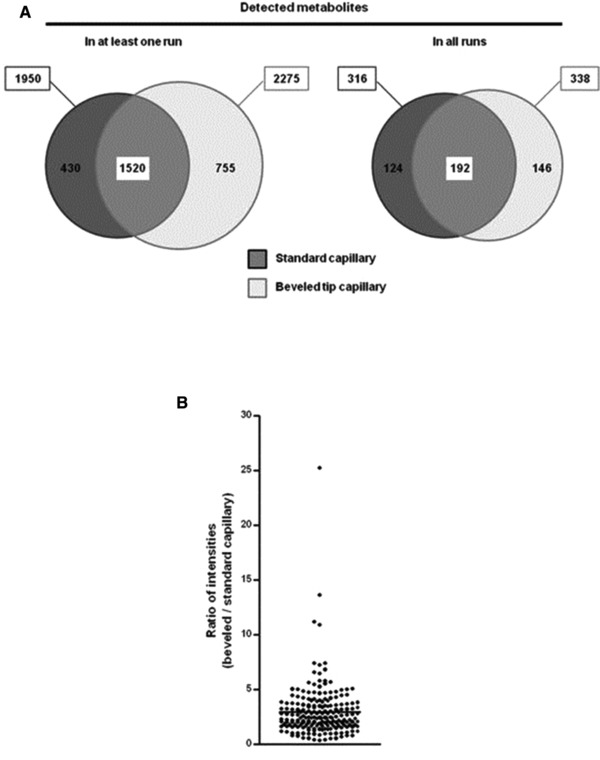
Comparison of CE‐MS using a beveled tip interface versus a CE‐MS approach using a sheath‐liquid interface for the analysis of a pooled human urine sample (*n* = 10). (A) Euler diagrams showing for each CE‐MS approach the number of metabolite features detected at least once (left) or every time (right). Dark gray: standard (flat tip) capillary; light gray: beveled tip capillary. (B) For each metabolite detected in every run and with both types of capillaries (*n*  =  192), the mean intensity was calculated and then the ratio between intensity measured with beveled tip capillary and intensity measured with classical capillary was calculated. Graph shows the mean ratio ± SEM, indicating that metabolite detection was more sensitive with beveled tip than with standard capillary. Reproduced from 9 with permission.

In order to improve the detection sensitivity of CE‐MS for urinary metabolic profiling studies, Boizard et al. used a beveled tip sheath‐liquid interface, which was first developed by Tseng et al. 36, instead of a conventional sheath‐liquid interface 9. When applied to the analysis of pooled human urine samples, this CE‐MS approach provided the detection of slightly more metabolite features as compared to the standard CE‐MS method (Fig. 5A). However, when for each metabolite observed in every run by both approaches, the mean intensity was determined and subsequently the ratio between intensity obtained with beveled tip CE‐MS and intensity obtained with classical CE‐MS was calculated, on average a threefold improvement in sensitivity was provided by the beveled tip CE‐MS approach (Fig. 5B). Moreover, the detection sensitivity of this approach was not affected after 50 runs, as was the case for sheath‐liquid CE‐MS. Actually, the beveled tip CE‐MS approach could be used for reproducible metabolic profiling of pooled urine samples over a range of four years. Therefore, this study clearly also demonstrates the potential usefulness of CE‐MS for metabolic profiling of large cohorts of urine samples.

Variability of migration times is an important issue in comparative metabolic profiling studies. In contrast to chromatographic‐based separation techniques, (open‐access) software tools for effectively correcting shifts in migration times are lacking, while such a tool is highly needed to improve the overall data robustness of CE‐MS for metabolomics and to further complete the analytical workflow. In this context, González‐Ruiz et al. has developed a new software tool, introduced as ROMANCE, for converting migration times into effective electrophoretic mobilities, thereby correcting for the shifts in migration times mainly resulting from variations in the magnitude of the EOF 37. Basically, this software tool converts the migration time scale into an effective electrophoretic mobility scale. Overall, it is expected that this approach will be of great value for (comparative) metabolic profiling studies. Moreover, the use of effective electrophoretic mobilities will be of high value for the identification of metabolites in biological samples, especially when using libraries based on electrophoretic mobilities.

A major bottleneck in metabolomics remains the identification of unknown metabolites of biological/clinical significance, especially when standards do not exist or MS/MS spectra are not available within public databases. Therefore, Yamamoto et al. developed a metabolomics‐based chemo‐informatics strategy for ranking candidate structures of unidentified peaks generated by CE‐MS 38. The strategy is based on using information of known metabolites observed in the samples containing the unidentified peaks and consists of three steps. Step one is concerned with the identification of so‐called “precursor/product metabolites” as potential reactants or products derived from the unidentified peaks. In step two, candidate structures for the unidentified peak are searched against the PubChem database using a molecular formula. These structures are then categorized by structural similarity against precursor/product metabolites and candidate structures. In the final step, the migration time is predicted to further refine the candidate structures. For this, a model was constructed for anionic and cationic metabolites using a random forest regression approach, in which migration time was used as response variable, and net charge and molecular weight as explanatory variables. This approach was effectively used for the identification of two unknown peaks in a urine sample as glycocyamidine and N‐acetylglycine. Though promising, the utility of the proposed strategy needs further verification by the identification of more unknown peaks in a given biological sample.

## 3 Applications

The applicability of CE‐MS for metabolomics in various fields was demonstrated in 43 publications in the period from July 2016 to June 2018. The search terms “metabolomics,” “metabolic profiling,” “metabolic fingerprinting,” “capillary electrophoresis and MS” were used for selecting these studies from ISI Web of Science and PubMed databases. An overview of these studies is given in Table 1, which provides information about the type of sample and compounds analyzed, the BGE, sample pretreatment procedure, the MS analyzer employed, LOD (when provided by the authors), and the type of capillary coating used. In the following sections, representative examples of CE‐MS‐based metabolomics are discussed.

**Table 1:** Overview of CE‐MS‐based metabolomics studies reported between July 2016 and June 2018

Compounds	Sample matrix	BGE	Sample pretreatment	MS analyzer	LOD^a^	Remarks	Ref.
Anionic and cationic metabolites	Human plasma	50 mM ammonium acetate (pH 8.5); 1 M formic acid (pH 1.8)	Methanol/water/chloroform extraction; methanol/water layer evaporated; dried extract reconstituted in water	TOF	n.s.	Internal standards for quantification	7, 8
Cationic metabolites	Human urine	250 mM formic acid containing 20% acetonitrile	Urine diluted with 2 M urea, 0.0125% ammonium hydroxide, 100 mM NaCl and 0.01% SDS; ultrafiltration with 20‐kDa filter; filtrate applied to gel filtration column; eluate lyophilized and reconstituted in water	TOF	n.s.	Beveled tip sheath‐liquid interface	9
Anionic metabolites	Human urine	50 mM ammonium bicarbonate (pH 8.5)	Urine centrifugated; supernatant diluted in water (1:5, v/v)	TOF	n.s.	Hydrodynamic pressure gradient applied during separation	18
Cationic metabolites	*Xenopus laevis* embro	1% formic acid	Borosilicate capillary for extraction cell content; metabolites extracted with acetonitrile, methanol, and water (2:2:1, v/v).	TOF	low nM‐range	Home‐made microflow sheath‐liquid interface	33, 50
Cationic metabolites	Human colorectal adenocarcinoma cells	10% acid acid (pH 2.2)	Methanol/water/chloroform extraction; methanol/water layer evaporated; dried extract reconstituted in water	TOF	30–1000 nM	Sheathless interface	34
Anionic and cationic metabolites	HEK 293T cells	10% acetic acid (pH 2.2)	Cell pellet snap‐frozen with liquid nitrogen; cell debris removed with centrifugation; supernatant evaporated; dried extract reconstituted in water	TOF	n.s.	Low‐pH BGE for anionic metabolic profiling; no nebulizing gas applied; anionic metabolites detected as ammonium adducts or as protonated compounds in positive ion mode; a positive pressure of 30 mbar was applied at the CE inlet	35
Cationic metabolites	Human plasma	10% acid acid (pH 2.2)	Acetonitrile for protein precipitation followed by centrifugation; supernatant evaporated; dried extract reconstituted in water	TOF	n.s.		37
Anionic and cationic metabolites	Human urine	10% acid acid (pH 2.2)	Urine diluted in water (1:5, v/v); ultrafiltration using 5‐kDa filter; filtrate diluted with water (1:5, v/v)	TOF	n.s.		38
Cationic metabolites	Human urine	Formic acid, methanol and water at ratio 0.5/50/49.5	Ultrafiltration using 15‐kDa filter; sarcosine analysis required solid‐phase extraction	Triple quadrupole	n.s.	Flow‐through microvial interface	39
Anionic and cationic metabolites	Human saliva	50 mM ammonium acetate (pH 8.5); 1 M formic acid (pH 1.8)	Ultrafiltration using 5‐kDa filter	TOF	n.s.	Internal standards for quantification	40
Anionic and cationic metabolites	Human saliva and tissue samples	50 mM ammonium acetate (pH 8.5); 1 M formic acid (pH 1.8)	Methanol/water/chloroform extraction for tissues; methanol/water layer evaporated; dried extract reconstituted in water; Ultrafiltration using 5‐kDa filter for saliva	TOF	n.s.	Internal standards for quantification	41
Cationic metabolites	Aqueous humor	0.8 M formic acid containing 10% methanol	Dilution with water (1:5, v/v)	TOF	n.s.		42
Cationic metabolites	Human plasma	5 M acetic acid	Dithiothreitol and acetonitrile for protein precipitation; iodoacetic acid applied to prevent oxidation of thiols.	TOF	35–268 nM		43
Anionic and cationic metabolites	Human sweat	50 mM ammonium carbonate (pH 8.5); 1 M formic acid (pH 1.8) containing 15% acetonitrile	No sample pretreatment	TOF	n.s.	Multisegment injection approach	44
Anionic and cationic metabolites	Dried blood spots	50 mM ammonium carbonate (pH 8.5); 1 M formic acid (pH 1.8) containing 15% acetonitrile	Methanol for protein precipitation; supernatant centrifugated using 3‐kDa filter; filtrate evaporated and reconstituted in water	TOF	low nM‐range for cationic metabolites; low μM‐range for anionic metabolites	Multisegment injection approach	46
Anionic and cationic metabolites	Tobacco leaves	50 mM ammonium acetate (pH 8.5); 1 M formic acid (pH 1.8)	Methanol/water/chloroform extraction; methanol/water phase filtered with 5‐kDa ultrafiltration membrane followed by evaporation and reconstitution in water	TOF	n.s.	Internal standards for quantification	47
Anionic metabolites	Rice	50 mM ammonium carbonate (pH 8.5)	Methanol/water/chloroform extraction; methanol/water phase filtered with 5‐kDa ultrafiltration membrane followed by evaporation and reconstitution in water	TOF	n.s.	Internal standards for quantification	48
Anionic and cationic metabolites	Mouse colonic tissue, portal and cardiac blood	50 mM ammonium acetate (pH 8.5)	Methanol/water/chloroform extraction; methanol/water phase filtered with 5‐kDa ultrafiltration membrane followed by evaporation and reconstitution in water	TOF	n.s.	Internal standards for quantification	49
Anionic and cationic metabolites	Human plasma	50 mM ammonium acetate (pH 8.5); 1 M formic acid (pH 1.8)	Methanol/water/chloroform extraction; methanol/water layer evaporated; dried extract reconstituted in water	TOF	n.s.	Internal standards for quantification	51, 52
Anionic and cationic metabolites	Human saliva	50 mM ammonium acetate (pH 8.5); 1 M formic acid (pH 1.8)	Ultrafiltration using 5‐kDa filter	TOF	n.s.	Internal standards for quantification	53, 54
Anionic and cationic metabolites	Mouse skeletal cells	50 mM ammonium acetate (pH 8.5); 1 M formic acid (pH 1.8)	Methanol/water/chloroform extraction; methanol/water layer evaporated; dried extract reconstituted in water	TOF	n.s.	Internal standards for quantification	55
Cationic metabolites	Human plasma and serum		Formic acid/acetonitrile extraction; methanol/water layer evaporated; dried extract reconstituted in water	TOF	n.s.		56, 58
Anionic and cationic metabolites	Human serum and plasma	50 mM ammonium acetate (pH 8.5); 1 M formic acid (pH 1.8)	Methanol/water/chloroform extraction; methanol/water layer evaporated; dried extract reconstituted in water	TOF	n.s.		57, 62
Anionic and cationic metabolites	*In vitro* cell lines and mouse tissue	50 mM ammonium acetate (pH 8.5); 1 M formic acid (pH 1.8)	Methanol/water/chloroform extraction; methanol/water layer evaporated; dried extract reconstituted in water	TOF	n.s.		59
Anionic and cationic metabolites	Mouse feces and plasma	50 mM ammonium acetate (pH 8.5); 1 M formic acid (pH 1.8)	Methanol/water/chloroform extraction; methanol/water layer evaporated; dried extract reconstituted in water	TOF	n.s.		60
Anionic and cationic metabolites	Human saliva	50 mM ammonium acetate (pH 8.5); 1 M formic acid (pH 1.8)	Ultrafiltration using 5‐kDa filter	TOF	n.s.	Cationic coated capillary for anionic metabolic profiling	61
Anionic and cationic metabolites	Rat glioma tissues	50 mM ammonium acetate (pH 8.5); 1 M formic acid (pH 1.8)	Methanol/water/chloroform extraction; methanol/water layer evaporated; dried extract reconstituted in water	TOF	n.s.		63
Anionic and cationic metabolites	Induced pluripotent stem cells and embryonic cells	50 mM ammonium acetate (pH 8.5); 1 M formic acid (pH 1.8)	Methanol/water/chloroform extraction; methanol/water layer evaporated; dried extract reconstituted in water	TOF	n.s.		64
Anionic and cationic metabolites	Vascular tissues from rabbits	50 mM ammonium acetate (pH 8.5); 1 M formic acid (pH 1.8)	Methanol/water/chloroform extraction; methanol/water layer evaporated; dried extract reconstituted in water	TOF	n.s.		65
Anionic and cationic metabolites	Bovine aortic endothelial cells	50 mM ammonium acetate (pH 8.5); 1 M formic acid (pH 1.8)	Methanol/water/chloroform extraction; methanol/water layer evaporated; dried extract reconstituted in water	TOF	n.s.		66
Anionic and cationic metabolites	Primary hepatocytes	50 mM ammonium acetate (pH 8.5); 1 M formic acid (pH 1.8)	Methanol/water/chloroform extraction; methanol/water layer evaporated; dried extract reconstituted in water	TOF	n.s.		67
Anionic and cationic metabolites	Human renal carcinoma cells	50 mM ammonium acetate (pH 8.5); 1 M formic acid (pH 1.8)	Methanol/water/chloroform extraction; methanol/water layer evaporated; dried extract reconstituted in water	TOF	n.s.		68
Cationic metabolites	Breast tissue	0.5% acetic acid	Extraction with cold acetonitrile‐water (8/2, v/v); Supernatant centrifugated, filtrate evaporated and reconstituted in water	OrbiTrap velos	n.s.	Electrokinetic sheath‐liquid interface	69
Cationic metabolites	Liver tissue from Wistar rats	0.8 M formic acid containing 10% methanol	Formic acid/acetonitrile extraction; supernatant ultrafiltrated with 30 kDa‐filter	TOF	n.s.		70
Cationic metabolites	*Leishmania donovani*	0.8 M formic acid containing 10% methanol	Formic acid/acetonitrile extraction; supernatant ultrafiltrated with 30 kDa‐filter	TOF	n.s.	Lead selection of drugs	71
Anionic and cationic metabolites	*Orostachys japonicus* A. Berger (herb)	50 mM ammonium acetate (pH 8.5); 1 M formic acid (pH 1.8)	Methanol/water/chloroform extraction; methanol/water layer evaporated; dried extract reconstituted in water	TOF	n.s.		72
Anionic and cationic metabolites	Tumour tissue extracts of patients with color rectal cancer	50 mM ammonium acetate (pH 8.5); 1 M formic acid (pH 1.8)	Methanol/water/chloroform extraction; methanol/water layer evaporated; dried extract reconstituted in water	TOF	n.s.		73

a) LOD = limit of detection (S/N = 3); ns, not specified in paper.

### 3.1 Biomedical and clinical applications

**Figure 6:**
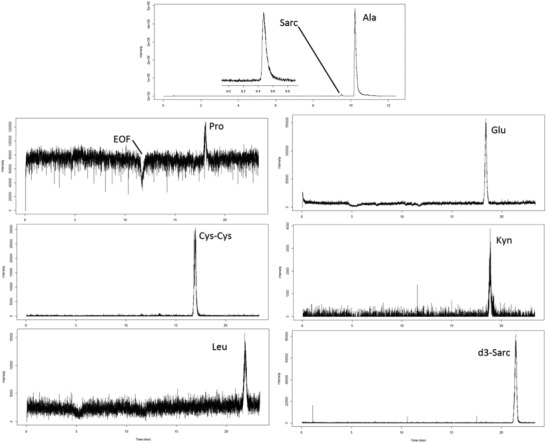
Extracted ion electropherograms obtained for the analysis of sarcosine and some other amino acids urine of a prostate cancer patient by CE‐MS using a flow‐through microvial interface. Electrophoretic separation at –30 kV performed with cationic polymer‐coated capillary using a BGE of formic acid, methanol, and water (0.5/50/49.5, v/v/v); sample injection, 1 psi for 10 s. Reproduced from 39 with permission.

MacLennan et al. developed a CE‐MS method for both targeted and nontargeted profiling of cationic metabolites in urine samples of genitourinary cancer patients (prostate and/or bladder) 39. Electrophoretic separations in reversed polarity mode were carried out with a capillary coated with the cationic polymer trimethoxysilylpropyl polyethyleneimine using a BGE composed of formic acid, methanol, and water (0.5/50/49.5, v/v/v). A flow‐through microvial interface was used for coupling CE to MS, in which the sheath‐liquid solution (which had the same composition as the BGE) was provided at a flow‐rate of 300 nL/min. Targeted analysis was focused on the quantification of endogenous levels of sarcosine, which was enriched by solid‐phase extraction, and five other amino acid metabolites implicated in the progression of prostate cancer in four patients and in pooled urine samples from healthy subjects. Fig. 6 shows the results obtained for a urine sample of one of the patients. The same CE‐MS method was also used for nontargeted metabolic profiling (*m/z* 50–850) of patient urine samples, resulting in the detection of more than 400 molecular features. Principal component analysis revealed a clear distinction between urine samples from healthy subjects and from prostate cancer patients. However, the number of patients in this study was too small to make any correlations between metabolite levels and progression of cancer.

Human saliva has become an attractive body fluid for disease prediction and diagnosis, as it can be collected in a noninvasive manner. Moreover, saliva testing is relatively simple, safe, and a low‐cost procedure. However, in order to be useful as a source for finding potential biomarkers of diseases, various confounding factors need to be characterized, such as the effect of saliva collection time after consumption of a meal. For this purpose, Ishikawa et al. used a sheath‐liquid CE‐MS approach to assess the effect of duration after consuming meals on the metabolic composition of saliva samples collected from oral cancer patients 40. Saliva from cancer patients was collected 12 h after dinner, and 1.5 and 3.5 h after breakfast. Control subjects fasted >1.5 h prior to saliva collection. Cationic metabolites were analyzed at low‐pH separation conditions, whereas anionic metabolites were analyzed at high‐pH separation conditions using a bare‐fused silica capillary and normal CE polarity in both cases. Levels of 51 metabolites differed significantly in controls versus oral cancer patients at the 12‐h fasting time point (*p* < 0.05). Fifteen and ten metabolites differed significantly at the 1.5‐ and 3.5‐h time points, respectively. The area of under receiver operating characteristic curve for discriminating oral cancer patients from controls was greatest at the 12‐h fasting time point. Overall, the CE‐MS‐based metabolomics study revealed that collection time after meals (significantly) affects levels of salivary metabolites for oral cancer screening. Therefore, such information is critical to include in saliva collection protocols for metabolomics‐based biomarker discovery studies.

Ishikawa et al. used a sheath‐liquid CE‐MS approach to find potential metabolic biomarkers for oral cancer screening in saliva and tumor tissue samples 41. Tumor and control tissues were obtained from oral cancer patients and saliva samples were collected from patients and healthy controls. Cationic metabolites were analyzed at low‐pH separation conditions, whereas anionic metabolites were analyzed at high‐pH separation conditions using a bare‐fused silica capillary and normal CE polarity in both cases. Protocols from HMT were employed for the extraction of metabolites from saliva and tumor tissue samples. The CE‐MS‐based metabolomics study revealed that the levels of 85 metabolites were significantly different between tumor and matched control samples, whereas 45 metabolites were significantly different between saliva samples from oral cancer patients and controls (*p* < 0.05 correlated by false discovery rate). Seventeen metabolites showed consistent differences in both saliva and tissue‐based comparisons, of which a combination of only two metabolites provided a high area under receiver operating characteristic curves (0.827; 95% confidence interval, 0.726–0.928, *p* < 0.0001) for discriminating oral cancer patients from controls. However, no significant differences in disease stages and histological types were identified, thereby preventing their usefulness for the screening of oral cancer.

Barbas‐Bernardos et al. developed a CE‐MS method for metabolic profiling of aqueous humor, i.e. the transparent fluid found in the anterior chamber of the eye, in order to assess the utility of this body fluid for providing new biochemical insights in ocular diseases, in this case myopia 42. Metabolic profiling by CE‐MS was performed with a fused‐silica capillary using a BGE of 0.8 M formic acid containing 10% methanol. A total of 44 cationic metabolites were provisionally identified in aqueous humor by this method using only a low amount of sample. In order to obtain a wider metabolic coverage for biomarker discovery, CE‐MS was combined with RP‐LC‐MS to study groups of patients with high and low myopia. CE‐MS analysis provided 5 compounds (mostly amino acids), whereas analysis by RPLC‐MS yielded 17 compounds (mostly lipids) in aqueous humor for selectively distinguishing patients based on the severity of myopia. Amino‐octanoic acid, arginine, citrulline, and sphinganine were highly abundant in patients with high myopia, whereas patients with low myopia had high levels of amino‐undecanoic acid, dihydro‐retinoic acid, and cysteinylglycine disulfide. Overall, this study provided useful information about the metabolic composition of human aqueous humor and myopia.

Cieslarova et al. developed a CE‐MS approach for the determination of homocysteine, cysteine, methionine, and glutamic acid in human plasma in order to assess the role of these metabolites as potential biomarkers for amyotrophic lateral sclerosis (ALS) 43. Sample preparation was carefully evaluated as sulfur‐containing amino acids may interact with plasma proteins. Dithiothreitol and acetonitrile were used for plasma protein depletion. To prevent the oxidation of thiols to disulfide thiol groups, iodoacetic acid was used before determination of homocysteine and cysteine by CE‐MS. All amino acids were analyzed by CE‐MS using 5 M acetic acid as BGE and 5 mM acetic acid in methanol/water (1:1, v/v) as sheath liquid. LODs in the range from 35 nM (homocysteine) to 268 nM (glutamic acid) were obtained by this approach, which were sufficiently low to allow the quantification of these compounds in plasma samples. The method was applied to the analysis of plasma samples from a group of healthy individuals (*n* = 20) and patients with ALS (*n* = 39). Significantly higher concentrations of glutamic acid and cysteine were found in plasma of ALS patients, however, to assess their usefulness as potential biomarkers for ALS the study needs to be performed with large sample cohorts.

**Figure 7:**
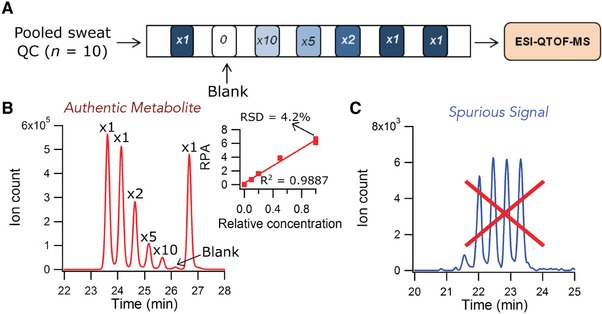
Dilution trend filter for nontargeted metabolic profiling of the sweat metabolome based on temporal signal pattern recognition using CE‐MS with multisegment injection. (A) Injection configuration for the dilution trend filter using a pooled sweat QC (*n* = 10) serially diluted by factors of 1, 2, 5, and 10‐fold, including a triplicate for the least diluted sample and a blank. (B) Example extracted ion electropherogram (EIE) of an authentic feature (citrulline, *m/z* 176.1030, ESI+), which follows the dilution trend (*R* ^2^ = 0.989), can be reliably measured with good precision (RSD = 4.2%, *n* = 3), and shows no background signal in the blank (i.e., signal is derived from sweat). (C) Example of a spurious signal (*m/z* 178.1588, ESI+), which does not follow the expected dilution trend and can be confidently excluded from the mass list. Reproduced from 44 with permission.

Recently, Macedo et al. developed a CE‐MS approach for the characterization of the sweat metabolome from screen‐positive cystic fibrosis (CF) infants with the aim to identify metabolites that are associated with CF disease status in order to complement sweat chloride testing 44. In this study, pilocarpine‐stimulated sweat samples were collected independently from two CF clinics, including 50 unaffected infants and 18 confirmed CF cases. Metabolic profiling by CE‐MS was performed using a bare fused‐silica and a BGE of 1 M formic acid (pH 1.8) containing 15% acetonitrile for cationic metabolites, while a BGE of 50 mM ammonium bicarbonate (pH 8.5) was used for profiling anionic metabolites. A conventional sheath‐liquid interface was used for coupling CE to MS. To increase sample throughput, a multisegment injection (MSI) approach was employed, which was previously developed by the same group 45. As shown in Fig. 7, authentic metabolite features in sweat could be readily annotated based on their temporal signal pattern when using the MSI approach in combination with high resolution tandem mass spectrometry. Amino acids, organic acids, amino acid derivatives, dipeptides, purine derivatives, and unknown exogenous compounds were identified in sweat, including metabolites associated with affected yet asymptomatic CF infants, such as asparagine and glutamine. The CE‐MS‐based metabolomics study revealed that pilocarpic acid, a metabolite of pilocarpine (used to stimulate sweat secretion in infants), and a plasticizer metabolite from environmental exposure, mono(2‐ethylhexyl)phthalic acid, were secreted in the sweat of CF infants at significantly lower concentrations as compared to unaffected CF screen‐positive controls. These findings indicated a deficiency in human paraoxonase, an enzyme not related to mutations to the cystic fibrosis transmembrane conductance regulator and impaired chloride transport, which is a nonspecific arylesterase/lactonase known to mediate inflammation, bacterial biofilm formation, and recurrent lung infections in affected CF children later in life. Hence, this metabolomics study provided new insights into the underlying mechanisms of CF pathophysiology. When the same CE‐MS metabolomics approach was applied to dried blood spots for the screening of inborn errors of metabolism, new biomarkers for the early detection of galactosemia, such as N‐galactated amino acids, were discovered 46. Methanol containing an internal standard was used for the extraction of metabolites from a filtered dried blood spot (3.2 mm or circa 3.4 μL).

### 3.2 Plant and microbial applications

**Figure 8:**
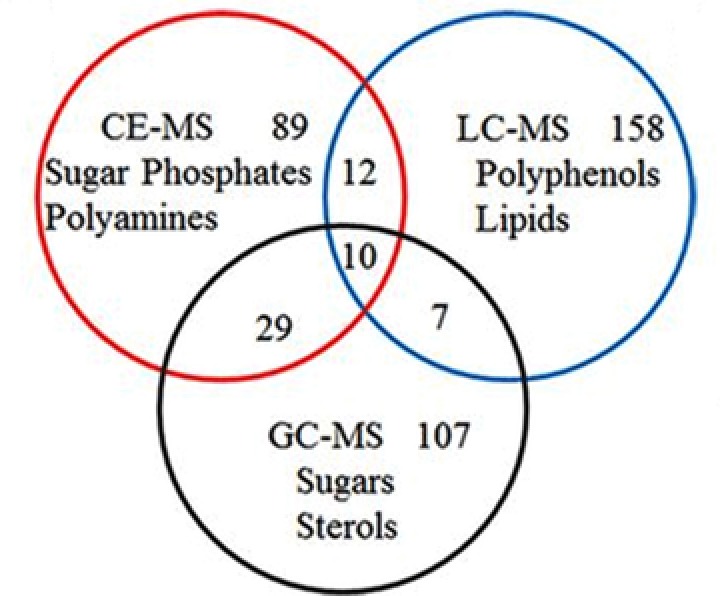
Metabolite classes specifically detected by CE‐MS, GC‐MS, and RPLC‐MS in extracts from tobacco leaves. Reproduced from 47 with permission.

In order to obtain insight into the biochemical processes underlying early senescence, which is a type of programmed cell death, in tobacco leaves, Li et al. developed a multianalytical platform comprised of CE‐MS, GC‐MS, and RPLC‐MS for comprehensive metabolic profiling of extracts from tobacco leaves 47. Metabolic profiling by CE‐MS was performed at low‐pH separation conditions for cationic metabolites and at high‐pH separation conditions for anionic metabolites. A bare‐fused silica capillary and normal CE polarity was used under both conditions. The reproducibility and stability of the CE‐MS method was assessed by analyzing quality control (QC) samples, which was a pool of all leave extracts. In total 140 metabolites were identified by CE‐MS in the QC sample, of which 97% had a RSD for peak areas below 30%. By using the multianalytical platform, 412 metabolites were identified in tobacco leave extracts. Figure 8 clearly shows the complementary value of CE‐MS for metabolomics, as highly polar and charged metabolites, such as sugar phosphates and polyamines, were mainly detected by this approach in tobacco leaves. These findings were in agreement with previous metabolomics studies in which CE‐MS was used as a complementary analytical tool in addition to RPLC‐MS, GC‐MS, and HILIC‐MS 5, 35. Comprehensive time‐dependent metabolic profiling was performed on tobacco middle leaves at five developmental stages (i.e., vigorous growth, 50% flower bud, full‐bloom, lower leaf ripening, and middle leaf ripening). Multivariate data analysis revealed that metabolic profiles of tobacco leaves were strongly affected by their developmental stages. With the onset of senescence, significant physiological changes followed, which activated or inhibited various metabolic pathways. Authors proposed follow‐up investigations in order to obtain a deeper understanding of the metabolic mechanisms.

*Rhizoctonia solani* is a fungal pathogen that causes sheath blight disease in rice plants. Suharti et al. developed a CE‐MS approach for the global profiling of anionic metabolites to investigate the resistance response of resistant and susceptible rice lines (32R and 29S, respectively) due to *R. solani* infection 48. Anionic metabolic profiling by CE‐MS was performed at high‐pH separation conditions using a bare fused‐silica capillary and electrophoretic separation was performed in normal polarity mode. The metabolomics study indicated that the two rice lines showed different responses to the infection of *R. solani*. In rice line 32R, *R. solani* infection induced significant increases in adenosine diphosphate, glyceric acid, mucic acid, and jasmonic acid. In rice line 29S, inosine monophosphate was involved in the plant response to *R. solani* infection. Overall, the study revealed different responses between the two rice lines in defense against *R. solani* infection.

Low‐molecular‐weight compounds produced by the intestinal microbiome play an important role in health and disease. However, little is known about the ability of the colon to absorb these metabolites. In order to obtain insight into this process, Matsumoto et al. used a CE‐MS‐based metabolomics approach, germ‐free (GF) mice, and colonized (Ex‐GF) mice to identify the colonic luminal metabolites transported to colonic tissue and/or blood 49. Metabolic profiling by CE‐MS was performed at low‐pH separation conditions for cationic metabolites and at high‐pH separation conditions for anionic metabolites. A bare‐fused silica capillary and normal CE polarity was used under both conditions. The metabolomics study focused on the differences in each metabolite between GF and Ex‐GF mice to determine the identities of metabolites that were transported to the colon and/or blood. CE‐MS identified 170, 246, 166, and 193 metabolites in the colonic feces, colonic tissue, portal plasma, and cardiac plasma, respectively. Overall, this study revealed for the first time the transportation of some metabolites from the colonic lumen to colonocytes and somatic blood in vivo, and these findings may be critical for obtaining a better understanding of host‐intestinal bacterial interactions.

Details of the remaining CE‐MS‐based metabolomics applications of the past two years can be found in Table 1 50, 51, 52, 53, 54, 55, 56, 57, 58, 59, 60, 61, 62, 63, 64, 65, 66, 67, 68, 69, 70, 71, 72, 73. In most studies, CE‐MS was used for the global profiling of metabolites in biological samples using bare fused‐silica capillaries. Low‐pH separation conditions were used for cationic metabolites with ESI‐MS detection in positive‐ion mode, whereas high‐pH separation conditions were used for anionic metabolites with ESI‐MS detection in negative ion mode. In general, a conventional sheath‐liquid interface was employed for coupling CE to MS. When different separation and/or detection conditions were utilized, this information is provided in Table 1.

## 4 Conclusions and perspectives

The utility of CE‐MS for metabolomics studies in various research fields was demonstrated in 43 publications over the past two years. The use of a CE‐MS‐based metabolomics approach provided useful insights into questions/problems from different fields. For example, new insights into the underlying mechanisms of CF pathophysiology was provided by global metabolic profiling of sweat samples by CE‐MS.

Within metabolomics, a strong asset of CE‐MS is its suitability for metabolic profiling of biological samples that are only available in limited amounts. This potential of CE‐MS has been recognized by multiple research groups over the past two years as many of the reported studies were focused on the analysis of volume‐limited samples, such as, saliva, sweat, dried blood spots, aqueous humor, and limited amounts of cells to even single cell analysis. Especially for the latter, CE‐MS has shown to be a very useful analytical technique 74, 75, 76. It is anticipated that CE‐MS will become a key technique for volume‐restricted metabolomics, especially with the use of sheathless interfacing designs.

Another visible trend is the use of CE‐MS as a complementary analytical technique when employing a multianalytical platform approach for comprehensive metabolomics studies. The studies highlighted in this paper clearly show the added value of CE‐MS in comparison to other analytical techniques for the profiling of highly polar and charged metabolites, notably for sugar phosphates, nucleotides, and amino acids.

Over the past few years, various research groups took a serious effort to assess the long‐term performance of CE‐MS for metabolomics studies and in the development of strategies for monitoring the performance of CE‐MS over time. In this context, the recent work from the group of Soga and coworkers, in which more than 8000 human plasma samples from the Tsuruoka Metabolomics Cohort Study have been analyzed over a 52‐month period with an acceptable reproducibility, is very encouraging. Overall, the studies reported here clearly shows the unique capabilities of CE‐MS for (volume‐restricted) metabolomics studies.

*Dr. Rawi Ramautar would like to acknowledge the financial support of the Vidi grant scheme of the Netherlands Organization of Scientific Research (NWO Vidi 723.016.003)*.

*The authors have declared no conflict of interest*.
